# Unleashing the potential of chatbots in mental health: bibliometric analysis

**DOI:** 10.3389/fpsyt.2025.1494355

**Published:** 2025-02-04

**Authors:** Qing Han, Chenyang Zhao

**Affiliations:** ^1^ Department of Information, Zhejiang Chinese Medical University, Hangzhou, China; ^2^ Department of Humanities, Zhejiang Chinese Medical University, Hangzhou, China

**Keywords:** chatbots, conversational agents, mental health, psychiatry, artificial intelligence, bibliometric, ChatGPT, large language models

## Abstract

**Background:**

The proliferation of chatbots in the digital mental health sector is gaining momentum, offering a promising solution to address the pressing shortage of mental health professionals. By providing accessible and convenient mental health services and support, chatbots are poised to become a primary technological intervention in bridging the gap between mental health needs and available resources.

**Objective:**

This study undertakes a thorough bibliometric analysis and discourse on the applications of chatbots in mental health, with the objective of elucidating the underlying scientific patterns that emerge at the intersection of chatbot technology and mental health care on a global scale.

**Methods:**

The bibliometric software Biblioshiny and VOSviewer were used to conduct a comprehensive analysis of 261 scientific articles published in the Web of Science Core Collection between 2015 and 2024. Publications distribution are analyzed to measure productivity on countries, institutions, and sources. Scientific collaboration networks are generated to analyze the influence as well as communications between countries and institutions. Research topics and trends are formulated by using a keyword co-occurrence network.

**Results:**

Over the last decade, researches on utilization of chatbots in mental health has appeared to be increasing steadily at an annual rate of 46.19%. The United States have made significant contributions to the development and expansion of publications, accounting for 27.97% of the total research output with 2452 citation counts. England came second to the US in terms of publications and citations, and followed by Australia, China, and France. National Center for Scientific Research in France ranked first among all institutions, followed by Imperial College London and University of Zurich. The number of articles published in Journal of Medical Internet Research was exceptionally high, accounting for 12.26% of the total number of articles, and JMIR Mental Health is the most influential publication sources in terms of average citations per article. Collaboration among universities in the USA, United Kingdom, Switzerland, and Singapore demonstrated a high level. The keyword co-occurrence network highlights the prominent techniques in this multidisciplinary area and reveals 5 research topics, showing a significant overlap between clusters. High-frequency terms such as “ChatGPT”, “machine learning”, and “large language models” underscore the current state of research, highlighting the cutting-edge advancements and frontiers in this field.

**Conclusions:**

This study provides an in-depth analysis of the most prominent countries, institutions, publications, collaboration status, and research topics associated with utilization of chatbots in mental health over the last decade. It offers insights to mental health professionals without an AI background and individuals interested in the development of mental health chatbots. The findings suggest that chatbots hold a significant role in promoting mental health well-being and exhibit considerable potential in demonstrating empathy, curiosity, understanding, and collaborative capabilities with users.

## 1 Introduction

Mental health issues affect approximately one billion individuals worldwide each year (1), with a notable rise in recent decades characterized by increased rates of suicidal behavior, substance misuse, and social isolation (2). As reported by the World Health Organization, one in four people will experience a mental health condition at some point in their lifetime (3). Specifically, depression and anxiety are the most prevalent mental health disorders globally, affecting an estimated 322 million and 264 million individuals, respectively (4).

Despite the escalating mental health demands, a global shortage of mental health professionals persists, with an unsustainable gap between demand and service provision (5). In the United States, approximately one in five adults experiences mental illness, yet many of these individuals do not access treatment (6). More than 75% of individuals in underdeveloped countries suffering from mental disorders receive no treatment (7). The scarcity of mental health resources hinders the implementation of individualized, evidence-based interventions, which are considered the gold standard in mental health treatment (8). The increasing public expectations for healthcare systems to deliver accessible, cost-effective, and evidence-based care to medically underserved populations is placing a significant strain on these systems, highlighting the need for innovative solutions to address the persistent mental health disparities (9).

Chatbots, commonly referred to as conversational agents, are sophisticated computer programs designed to mimic human conversation and are increasingly being integrated into diverse sectors, including customer service, healthcare, and e-commerce (10). The digital mental health space has witnessed a significant proliferation of chatbots, which have the potential to enhance accessibility to mental health services and support for a broader audience, thereby contributing to the alleviation of the existing mental health workforce shortage. Several chatbots, such as Woebot (11), have emerged as prominent providers of mental health assistance, offering users personalized advice and exercises grounded in cognitive behavioral therapy. Other chatbots, like Replika, possess the capacity to simulate emotional connections and psychological empathy, rendering them a valuable tool for health professionals as an adjunct to online therapies or patient monitoring (12). Furthermore, chatbots can guide users through structured exercises and interventions on mental health apps like Wysa and Tess (13). The efficacy of chatbots in addressing various mental health disorders, including depression, stress, and acrophobia, has been demonstrated (14), with applications extending to therapy, training, education, counseling, and screening within the mental health field (15). Additionally, chatbots play a pivotal role in promoting health through interventions such as digitally assisted mindfulness training in self-regulation skills for sustainable mental health (16), and breathing interventions for mental and emotional well-being (17).

The increasing utilization of chatbots in mental health has led to a notable surge in academic research exploring the intersection of artificial intelligence and mental health. This surge in research has enabled researchers to pinpoint knowledge and methodological gaps in the existing literature on chatbots and mental health. Alaa et al. (15) conducted a scoping review of patient perceptions and opinions regarding the use of chatbots for mental health care, resulting in the identification of 10 key themes, including usefulness, ease of use, responsiveness, understandability, acceptability, attractiveness, trustworthiness, enjoyability, content, and comparisons. A comprehensive review of digital mental health interventions was conducted by Eliane et al. (18), focusing specifically on AI-based chatbots and speculating on the future trajectory of AI in this field. The authors’ analysis highlighted the potential for chatbots to deliver personalized interventions, the evolution of AI capabilities, and the integration of dynamic mental health systems, hyper-personalization, and human-like interaction. Hannah (19) examined the use of conversational agent interventions in treating mental health issues and concluded that these interventions exhibit promising efficacy and acceptability. Ahmad et al. (20) undertook a systematic scoping review to assess the types of outcomes, outcome measurement instruments, and assessment methods employed in studies evaluating the effectiveness of conversational agent interventions for mental health. Their findings underscore the need for a standardized core outcome set and the adoption of validated instruments to ensure consistency and comparability across studies. Han et al. (21) carried conducted a systematic review to explore the potential of AI-based conversational agents in addressing mental health issues, synthesizing evidence on their effectiveness, user experience, and factors influencing their efficacy. However, these studies were limited by their narrow focus on specific mental health problems or scenarios, restricted search terms, and consequently identified a relatively small number of studies. Meanwhile, there is a scarcity of comprehensive bibliometric analysis on this research field. Because of its multidisciplinary foundations and diverse application areas, it is useful to have a clearer understanding of its status, trends, and topics across this research field.

In light of the existing knowledge gap in this research field, it is essential to conduct a comprehensive study focused on the investigation of chatbots in mental health, utilizing precise and exhaustive search terms. The primary objective of this study is to serve as a seminal resource for future research endeavors, contributing to the advancement of this field by providing a framework for future investigations. Bibliometric analysis offers a distinctive approach to evaluating global scientific output and identifying developmental trends, enabling researchers to gain a deeper understanding of the research landscape and facilitate interdisciplinary collaboration. As a quantifiable and objective method, bibliometrics enables the analysis of emerging trends and knowledge structures within a field, providing a robust foundation for data-driven decision-making (22). This study aims to contribute to the existing body of knowledge by providing a bibliometric analysis of scientific articles focused on the utilization of chatbots in mental health. Specifically, this study seeks to address the following research questions:

1. What are the primary attributes of the published articles?
2. Who are the most productive countries and institutes in these areas?
3. Which journals have published the highest number of articles?
4. How about the scientific collaborations between different countries?
5. What are the topics and trends in the research field of chatbots in mental health?

## 2 Methods

### 2.1 Search strategies

This study adhered to the Preferred Reporting Items for Systematic Reviews and Meta-Analysis (PRISMA) guidelines, which were selected as the preferred approach due to its rigorous guidelines to evaluate the existing literature and provide comprehensive insights (23). In this study, bibliographic data were extracted from the Web of Science Core Collection (WoSCC), a widely regarded and authoritative database, as it provides comprehensive coverage of multidisciplinary academic journals and is recognized for its high-quality publications. Only articles indexed in WoSCC were considered for inclusion, as it is a primary source of citation information and is widely regarded as an authoritative database among researchers, owing to its comprehensive collection of scholarly journals and its superior quality compared to other databases (24, 25).

The objective of this study is to investigate the utilization of chatbots in mental health, necessitating the explicit identification of the two central themes: “chatbots” and “mental health.” To inform our search strategy, we drew upon existing research on the intersection of chatbots and mental health to define the key search terms for each topic (8, 26, 27). Utilizing the WoSCC database, we conducted a comprehensive search for documents that include these terms in their titles, abstracts, or keywords. To enhance the specificity of our search, we employed the Boolean operator “OR” to capture variations of each term, and the operator “AND” to combine the two central themes. Additionally, we employed asterisks as wildcards to account for nuanced variations in the terminology. The search strategies employed in this study are detailed in **Table 1**.

**Table 1:** Query strategies and results.

Database	Query String	Query Time	Results
Web of Science Core Collection	TS=(“mental health” OR “mental illness*” OR “mental disorder*” OR “psychological health” OR “stress” OR “anxiety” OR “depression” OR “affective disorder*” OR “psychotic disorder*” OR “psychiatric disorder*” OR “depressive” OR “trauma” OR “insomnia” “sleep disorder*” OR “suicide*” OR “mental health treatment*” OR “mental health assessment*” OR “psychotherapy” OR “psychosis” OR “psychotic” OR “bipolar disorder*” OR “borderline personality disorder*” OR “schizophrenia”) AND TS=(“chatbot*” OR “chat bot” OR “virtual assistant” OR “conversational agent” OR “virtual agent” OR “conversational ai”) AND PY= (2014–2024)	2024.6.16	359

### 2.2 Inclusion and exclusion criteria

**Figure 1:**
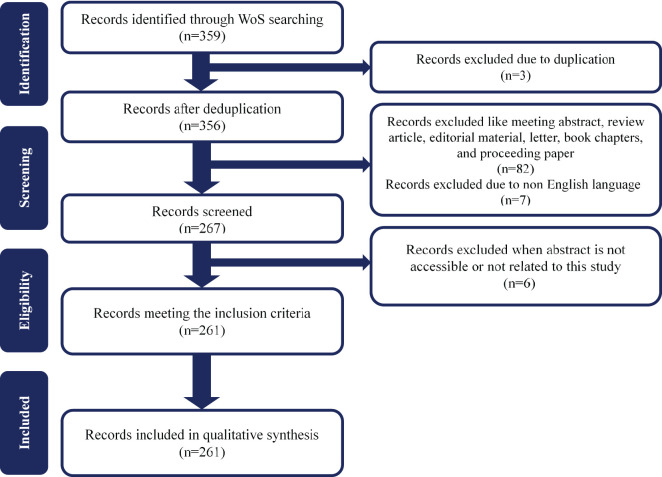
The flow diagram outlining the selection process used in this study.

Studies published since 2015 were included in our analysis, as this milestone represents the initial stages of chatbots’ integration into the mental health sector. Following the introduction of AI-based voice assistants to mobile phones and other Internet-of-Things devices by prominent technology companies, including Apple, Microsoft, Amazon, and Google, in 2015 (26), we established the study period from January 2015 to June 2024. **Figure 1** illustrates the flow diagram of the selection process. Two domain experts who have taught mental health for more than 10 years, first conducted the screening phrase independently, and then reached an agreement through discussion.

The following inclusion criteria were applied to the selected studies: (1) articles published in the English language, and (2) articles that focused exclusively on chatbots in the context of mental health research. Studies that did not specifically evaluate a particular chatbot’s efficacy in providing mental health support (e.g., protocols, meta-analyses, perspectives) were excluded from the analysis. Furthermore, Studies of mental health without the chatbot component were also excluded. To ensure the integrity and consistency of the bibliometric analysis, a rigorous selection process was employed, thereby excluding abstracts, review articles, editorial materials, letters, book chapters, and proceeding papers from consideration. The initial dataset comprised 366 records, and after careful filtering, the study ultimately included 261 articles published between 2015 and 2024.

### 2.3 Bibliometric analysis

Bibliometric analysis is conducted utilizing the open-source bibliometrix package in R (28), a powerful tool that facilitates data analysis and visualization. The biblioshiny application, an integral component of the bibliometrix package, enables users to import, clean, and organize bibliographic data, as well as perform a range of bibliometric analyses. Through its web-based interface, biblioshiny allows users to conduct analyses at three distinct levels, namely source-level, author-level, and document-level assessments. Furthermore, the application enables users to visually map the structures of knowledge in conceptual, intellectual, and social contexts. The rankings of countries, journals and institutions are determined based on the number of published articles.

Meanwhile, VOSviewer is used to visualize country collaboration and keyword co-occurrence networks. VOSviewer is a publicly available, freeware software application designed for the visualization and construction of bibliometric maps. The units of analysis in VOSviewer can be tailored to suit the specific focus of the study, allowing researchers to select from a range of options, including journals, publications, citations, authors, or countries (29). Keyword co-occurrence analysis is a method employed to elucidate the underlying conceptual structure of the field. In this context, keywords are typically drawn from “author keywords”, and co-word analysis presumes that words frequently co-occurring with one another are thematically interconnected. Similar to topic modeling technologies (30), the identification of clusters of co-occurring keywords provides insight into the topical foci or knowledge domains that have been explored in the literature on the utilization of chatbots in mental health over the past decade. The frequency and strength of co-occurrence between keywords can be quantified, with more frequent co-occurrences indicative of a stronger relationship between the two concepts (31). Our primary objective was to provide a comprehensive understanding of the existing literature on chatbots in the realm of mental health, with a view to elucidating the underlying knowledge structure, identifying key contributors, analyzing prevailing research trends, pinpointing the most productive countries and institutions, and highlighting areas of intense research activity.

## 3 Results

### 3.1 Publication trend and descriptive statistics

**Figure 2:**
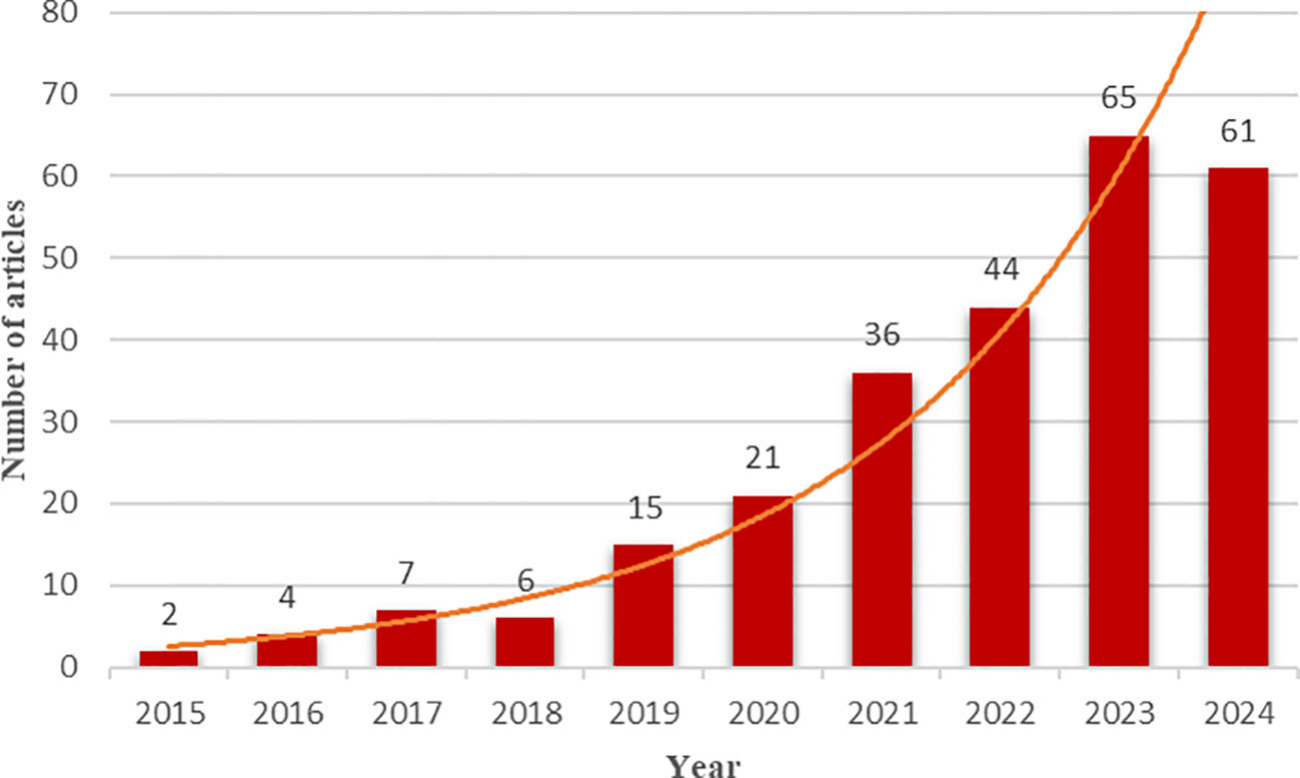
Number of articles published during the 2015-2024 period.

In **Figure 2**, we present an analysis of the yearly publication trends in the context of chatbot utilization in mental health. Notably, our data reveals a substantial increase in the number of publications over the years, with a notable rise from 2 articles in 2015 to 61 as of June 2024, demonstrating an annual growth rate of 46.19%. This field has been progressively developing during 2015 to 2018, experiencing a rapid growth since 2019. Specifically, the most significant growth happened in 2019 and 2021 with an increase in publications of 150% and 71.43% respectively. The main reason should be the outbreak of COVID-19. The COVID-19 pandemic, which began in 2019, has led to an escalation in recognized risk factors for mental health problems (32). During the COVID-19 pandemic, chatbots played a crucial role in addressing the pressing shortage of mental health professionals, particularly mental health advisors (33). A substantial body of research has been dedicated to exploring the utilization of chatbots in mental health. Over the past decade, the field has witnessed a significant expansion in terms of research output, indicating a substantial growth in the investigation and development of chatbot-based mental health solutions.

The final records comprise 261 full-text English articles cumulatively written by 1400 authors worldwide with 925 author’s keywords and published across 122 journals. **Table 2** presents the main statistics of the publications on utilization of chatbots in mental health for ten years, from 2015 to 2024. Notably, the analysis yielded substantial findings regarding co-authorship rates and international collaborations per document. These results suggest that authors within this field of research tend to favor collaborative endeavors, resulting in a high proportion of international partnerships, which is consistent with the prevailing trend of globalization in the mental health sector.

**Table 2:** Descriptive statistics of research on utilization of chatbots in mental health from 2015 to 2024.

Description	Results
MAIN INFORMATION ABOUT DATA	
Timespan	2015:2024
Sources (Journals)	122
Documents	261
Annual Growth Rate %	46.19
Document Average Age	2.13
Average citations per doc	18.44
References	12263
DOCUMENT CONTENTS	
Keywords Plus (ID)	618
Author’s Keywords (DE)	925
Authors	1400
Authors of single-authored docs	10
AUTHORS COLLABORATION	
Single-authored docs	10
Co-Authors per Doc	6.29
International co-authorships %	34.48

Source: Web of Science Core Collection.

### 3.2 Predominant countries and institutions

Top ten countries with the most publications are shown in **Table 3**. It compares the output of countries in terms of the number of journal articles and total publications. The results reveal that the top 10 countries, ranked by the number of journal articles published, are the United States, England, Australia, China, France, Germany, Netherlands, Spain, Singapore, and Canada, indicating a high level of scholarly engagement and publication activity among researchers from these nations.”

**Table 3:** Top 10 countries ranked by the article count.

Country	Period				Overall time		
	2015-2018		2019-2024		Scientific output		Citations
The United States	7	36.84%	66	27.27%	73	27.97%	2452
England	1	5.26%	33	13.64%	34	13.03%	898
Australia	0	0	21	8.68%	21	8.05%	537
China	0	0	21	8.68%	21	8.05%	213
France	4	21.05%	16	6.61%	20	7.66%	157
Germany	1	5.26%	19	7.85%	20	7.66%	230
Netherlands	4	21.05%	16	6.61%	19	7.28%	448
Spain	2	10.53%	16	6.61%	18	6.90%	197
Singapore	0	0	17	7.02%	17	6.51%	165
Canada	0	0	16	6.61%	16	6.13%	432
Total publications	19		242		261		

The United States emerged as the leading contributor to both lists, accounting for 27.97% of the total research output, with 2452 citation counts. In terms of publications, England ranked second to the United States, followed by Australia, Netherlands, Germany, and China in terms of citation counts. Notably, the presence of China and Singapore, the only two Asian countries featured on the lists, suggests a significant shift in research destinations away from the historically dominant Western nations.

As evident from **Table 3**, the initial stage of research on the utilization of chatbots in mental health was characterized by a limited body of literature, with the United States emerging as the dominant player, accounting for 36.84% of the total output. Conversely, Australia, China, Singapore, and Canada were absent from the top ten countries. However, a significant shift occurred in the subsequent stages. By 2019-2024, Australia and China had surpassed France and the Netherlands to become the third most productive countries in the field, with the Netherlands subsequently overtaking Germany to assume the second position in 2015-2018, only to be surpassed by Germany in 2020-2024. Furthermore, **Table 3** provides empirical evidence supporting the emergence of Australia, China, Singapore, and Canada as prominent research hubs in the utilization of chatbots in mental health from 2019-2024.

Based on the search results, nearly 200 institutions contributed to research on utilization of chatbots in mental health. **Table 4** presents a ranking of the top 10 most influential institutions within this research field, with the National Center for Scientific Research emerging as the leading institution, followed by Imperial College London and the University of Zurich.

**Table 4:** Top 10 institutions ranked by the article count.

Rank	Institutions	Country	Publications
1	National Center for Scientific Research	France	12
2	Imperial College London	England	11
3	University of Zurich	Switzerland	11
4	Nanyang Technological University	Singapore	10
5	National University of Singapore	Singapore	10
6	ETH Zurich	Switzerland	9
7	Stanford University	USA	9
8	Swiss Federal Institutes of Technology Domain	Switzerland	9
9	Harvard University	USA	8
10	University of Bordeaux	France	8

### 3.3 Productive publication sources

Regarding the sources, **Table 5** provides an overview of the most productive journals. Over the past decade, the Journal of Medical Internet Research has experienced an exceptionally high volume of publications, accounting for 12.26% of the total number of articles published during this period. Among the top 10 journals, 5 journals were cited more than 100 times. Internet Interventions, Journal of Medical Internet Research, and Frontiers in Psychiatry were the top 3 journals in terms of the average citations per article, 28.25, 20.97 and 9.38, respectively.

**Table 5:** Top 10 productive journals.

Rank	Sources	Articles	Citations	Average citations per article
1	Journal of Medical Internet Research	32	671	20.97
2	Frontiers in Psychiatry	13	122	9.38
3	JMIR Mental Health	11	1056	96
4	JMIR mHealth and uHealth	11	451	41
5	Digital Health	9	67	7.44
6	International Journal of Human-Computer Interaction	9	58	6.44
7	Internet Interventions	8	226	28.25
8	Behavior & Information Technology	6	6	1
9	ACM Transactions on Interactive Intelligent Systems	4	21	5.25
10	BMC Public Health	4	17	4.25

**Figure 3:**
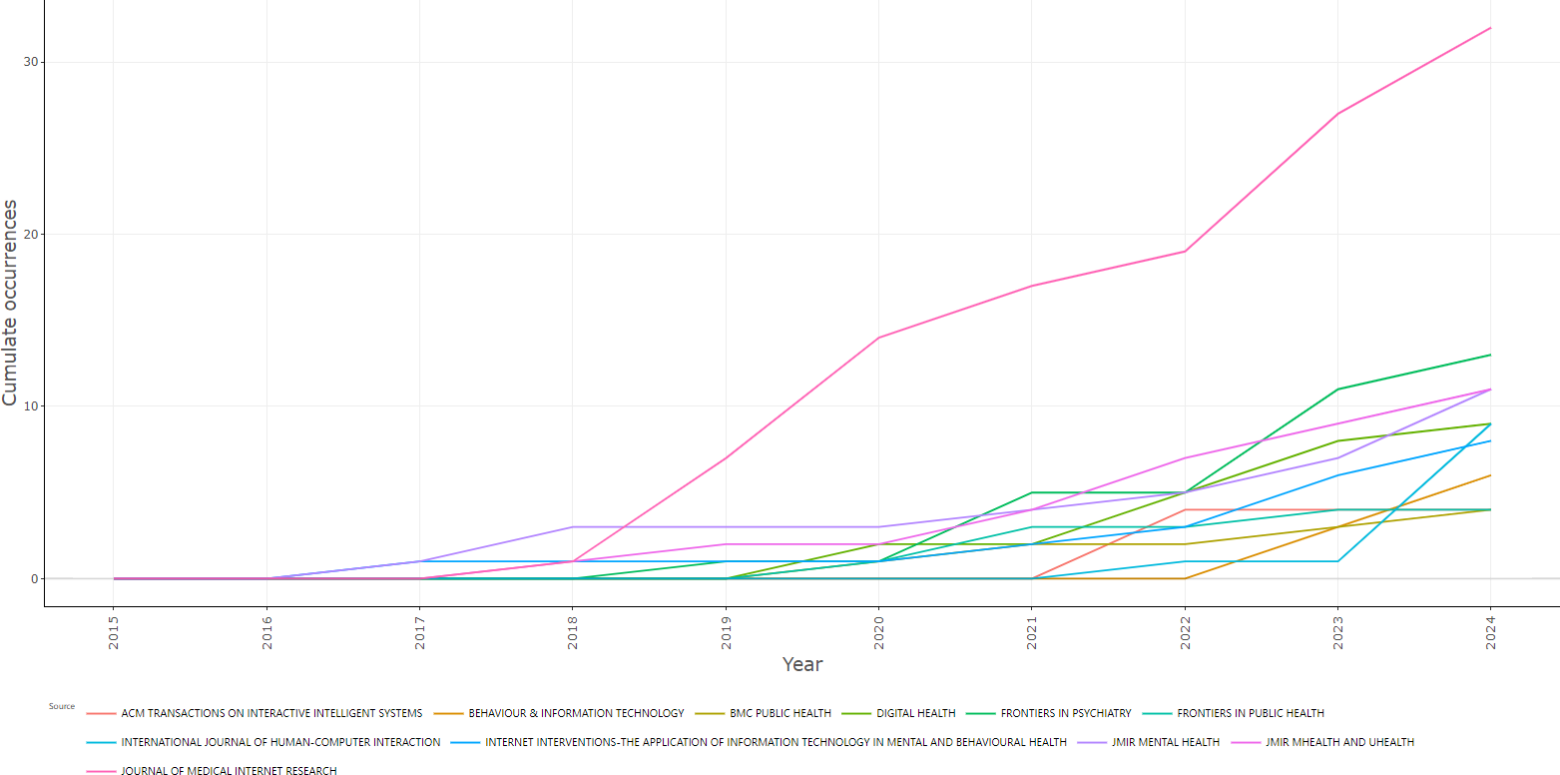
Source dynamics of the top 10 productive journals.

An examination of the source dynamics of the top 10 journals in this research field (as depicted in **Figure 3**) reveals a consistent upward trend in the cumulative publication volume. Specifically, the Journal of Medical Internet Research has demonstrated the most substantial increase in the number of publications within this domain over the period from 2019 to 2024.

### 3.4 Scientific collaboration networks

**Figure 4:**
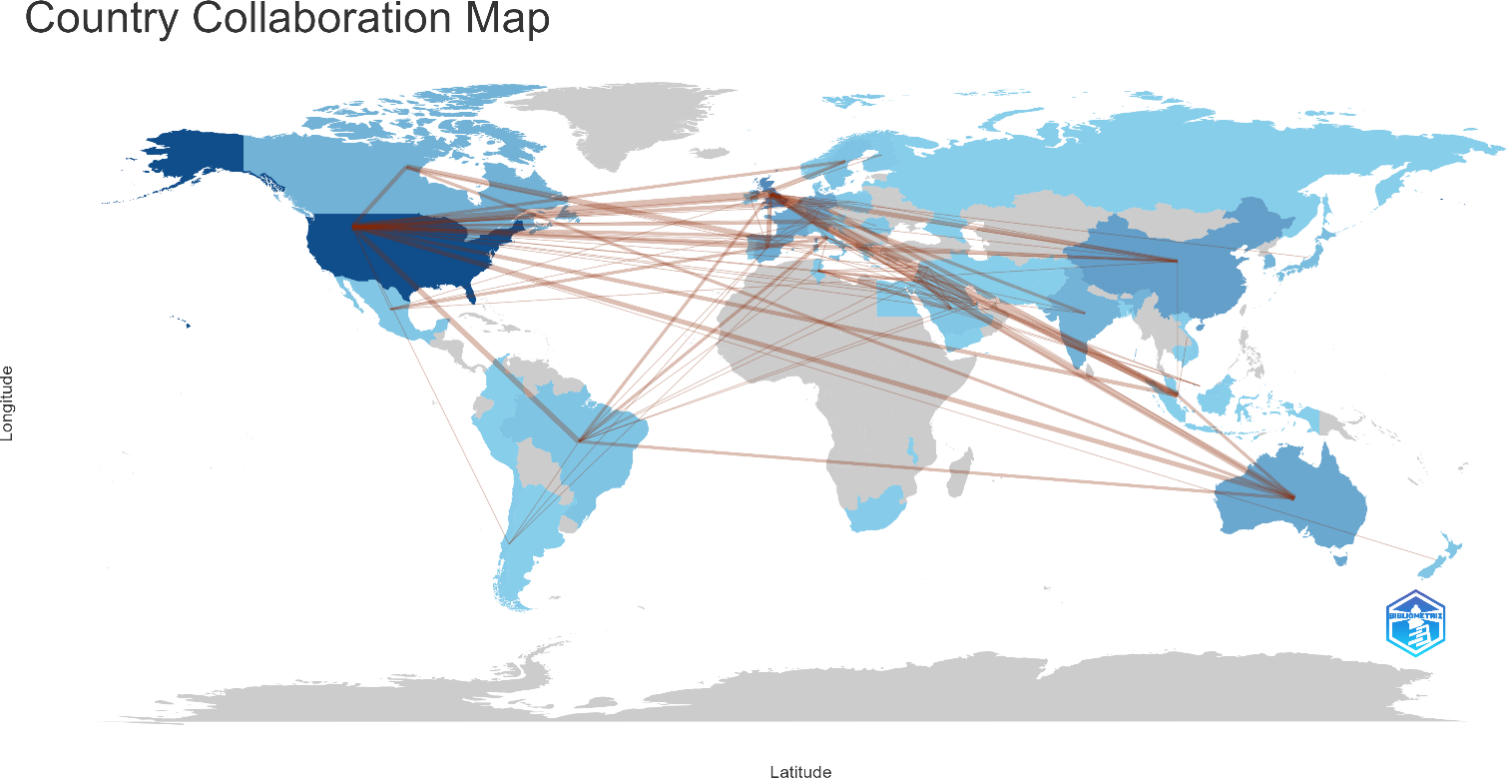
Country collaboration map of research on utilization of chatbots in mental health.

**Figure 5:**
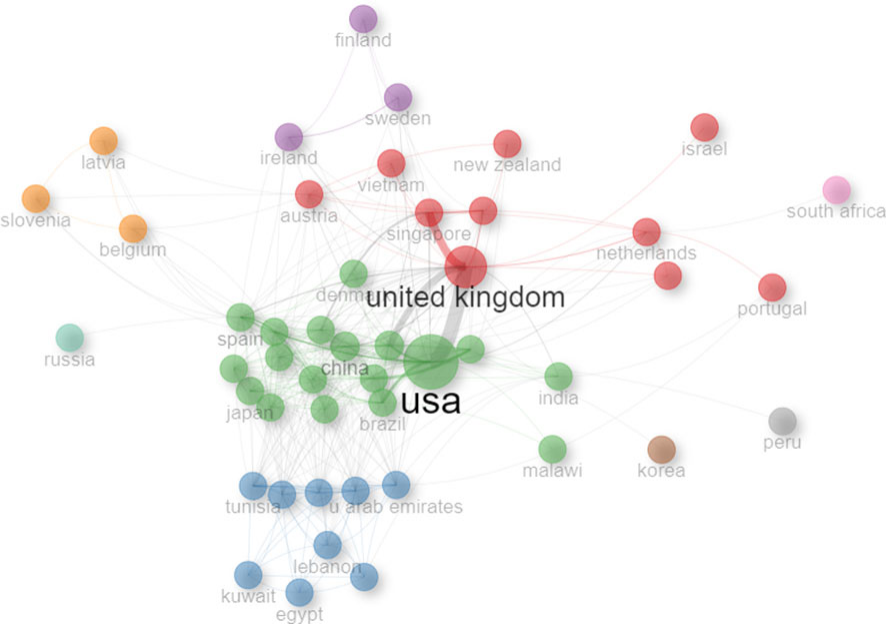
Collaboration network of countries.

A macro-level picture of how countries have collaborated on chatbots research in the field of mental health since 2015 is shown in **Figure 4**, suggesting that related research in both countries has benefited from international partnerships. The collaborative scientific research network among the countries is visualized in **Figure 5** using the Bibliometrix and biblioshiny. The top 10 scientific collaborations between two countries in terms of the frequency are shown in **Table 6**. USA with United Kingdom, United Kingdom with Singapore, and United Kingdom with Australia were the 3 closest partnerships in this research area. There is a pressing need for the establishment of standardized guidelines and responsible deployment of chatbots in the mental health sector on a global scale, owing to the potential risks associated with biases, data privacy concerns, and the dissemination of misinformation (34). This underscores the imperative of international cooperation in developing and implementing universally accepted standards and regulatory frameworks that prioritize the ethical and transparent use of mental health chatbots.

**Table 6:** Scientific collaborations between two countries.

Rank	From	To	Frequency
1	The United States	United Kingdom	13
2	United Kingdom	Singapore	10
3	United Kingdom	Australia	9
4	France	Canada	6
5	The United States	Brazil	6
6	Singapore	Switzerland	5
7	The United States	Australia	5
8	China	United Kingdom	4
9	Singapore	Germany	4
10	United Kingdom	Spain	4

**Figure 6:**
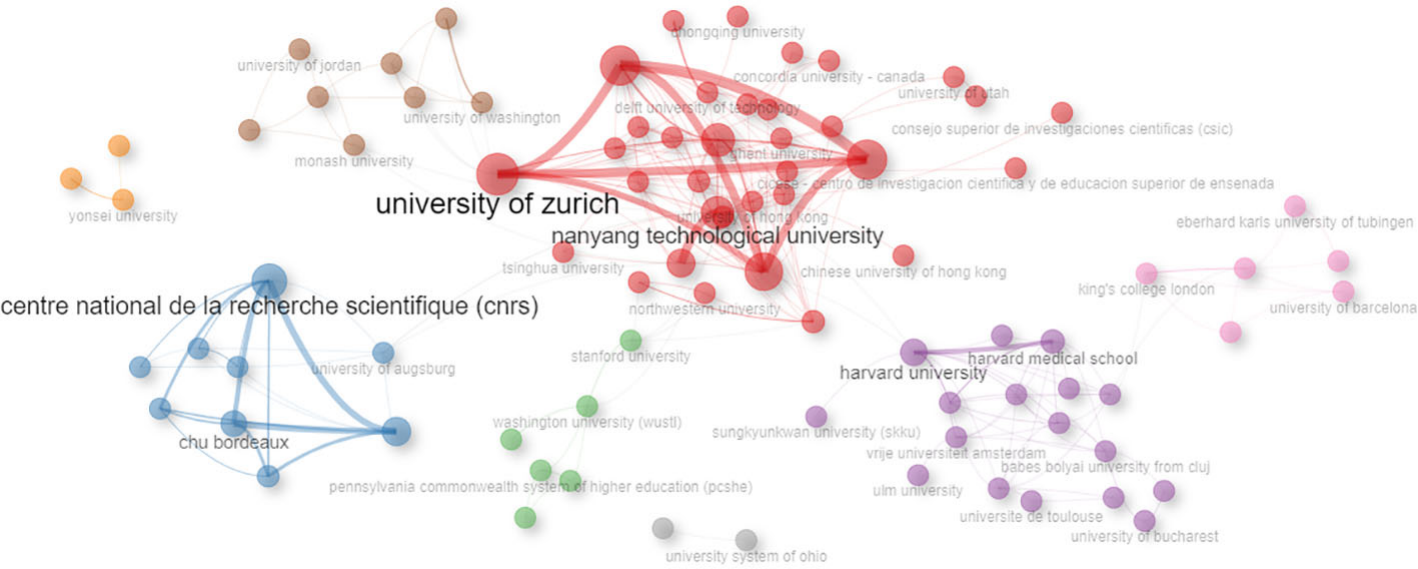
Collaboration network of institutions.

Collaboration network of institutions shows the influence as well as communications between institutions. The Bibliometrix and biblioshiny are used to generate the institution collaboration networks in this research field (**Figure 6**). The connections between institutions are determined through the analysis of co-authored publications, resulting in the formation of two discernible clusters, which are visually distinguished by color coding, thereby facilitating an understanding of the collaborative networks within this field of research. There are 7 clusters in the collaboration network of institutions when using the edge betweenness clustering algorithm. **Table 7** lists the top 10 institutions in terms of ‘Betweenness’ and ‘PageRank’ of the institution collaboration network. Nanyang Technological University, Harvard University, and University of Augsburg, and Washington University are the most influential institution in cluster 1, cluster 4, cluster 2, and cluster 3 respectively.

**Table 7:** Top 10 institutions in terms of ‘Betweenness’ of the institution collaboration network.

Rank	Node	Cluster	Betweenness	PageRank
1	Nanyang Technological University	1	1,428.598	0.026
2	Harvard University	4	1,299.229	0.029
3	University of Augsburg	2	568	0.007
4	University of Zurich	1	517.996	0.04
5	University of Melbourne	4	439.543	0.014
6	Imperial College London	1	399.703	0.017
7	Dartmouth College	1	386.667	0.013
8	Washington University in St. Louis	3	303	0.014
9	University of London	7	228	0.018
10	ETH Zurich	1	201.253	0.037

### 3.5 Research topics and trends

Keyword co-occurrence analysis offers a valuable approach to identifying emerging research topics within a scientific domain. Furthermore, a time-based visual analysis of keyword co-occurrence networks provides a comprehensive framework for examining the temporal dynamics of research trends. In this study, a keyword co-occurrence network was constructed from a dataset comprising 261 academic articles. Following a frequency threshold of 3 or greater, 88 prominent keywords were identified and selected for visualization. The size of the node corresponding to each keyword is proportional to its frequency of appearance, thereby facilitating the representation of research topics. The thickness of the connecting line between nodes serves as a proxy for the strength of association between the keywords; the greater the line’s thickness, the more frequently these keywords co-occur in the same article. Furthermore, distinct color coding is employed to differentiate between clusters, which correspond to distinct research topics.

**Figure 7:**
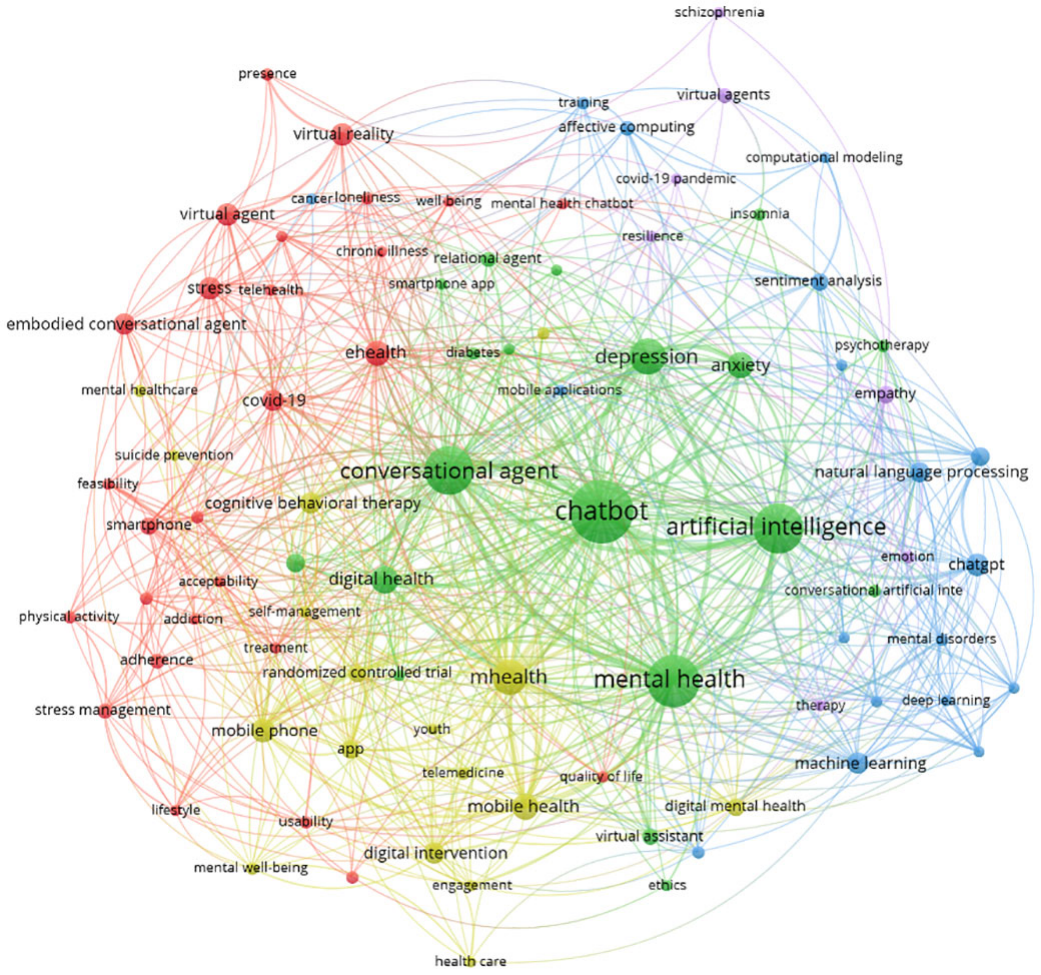
Keyword co-occurrence network.

**Figure 7** illustrates the keyword co-occurrence network and reveals 5 research topics in the field of utilization of chatbots in mental health from 2015 to 2024, showing a significant overlap between clusters. Details of clusters with their representative keywords are listed in **Table 8**.

1. The red cluster focuses on usability of chatbots for mental health. It involves keywords related to usability and mental health, such as acceptability, usability, user experience, embodied conversational agent, feasibility, smartphone, usability, virtual agent, virtual reality, etc., indicating that user experience is a crucial issue when using chatbots for mental health.
2. The green cluster extensively investigates utilization of chatbots in mental health. It includes keywords like anxiety, depression, diabetes, ethics, insomnia, mental health, obesity, psychiatry, psychotherapy, chatbot, body image, conversational agent, conversational ai, smartphone apps, virtual assistant, etc., suggesting that chatbots are widely used to solve various mental health problems.
3. The blue cluster revolves around the technologies used in chatbots for mental health. It encompasses keywords like machine learning, deep learning, natural language processing, predictive models, affective computing, computational modeling, large language model, etc., indicating that intelligent technologies are widely applied in chatbots.
4. The yellow cluster is dedicated to intervention, prevention and therapy of mental health problems. It comprises keywords like digital intervention, cognitive behavioral therapy, randomized controlled trial, suicide prevention, etc., indicating that chatbots are effective in various stages of clinical mental health problems.
5. The purple cluster centers its attention on mental health problems during pandemic periods. It includes keywords like covid-19, pandemic, emotion, resilience, therapy, virtual agents, etc., which collectively reflect the growing interest among researchers in leveraging chatbots as a tool for mitigating mental health concerns during pandemics.

**Table 8:** Clusters with their representative keywords.

Clusters	Keywords
The Red Cluster	Acceptability, addiction, adherence, ehealth, embodied conversational agent, feasibility, lifestyle, mental health chatbot, older adults, physical activity, psychoeducation, quality of life, smartphone, stress management, telehealth, treatment, usability, user experience, virtual agent, virtual reality, well-being
The Green Cluster	Adolescent, anxiety, artificial intelligence, body image, chatbot, conversational agent, conversational ai, depression, diabetes, digital health, ethics, insomnia, mental health, obesity, psychiatry, psychotherapy, relational agent, smartphone apps, virtual assistant
The Blue Cluster	Affective computing, ChatGPT, computational modeling, conversational user interface, deep learning, large language model, machine learning, medical treatment, mental disorders, mental illness, mobile applications, natural language processing, predictive models, sentiment analysis, training
The Yellow Cluster	App, artificial intelligence chatbot, cognitive behavioral therapy, digital intervention, digital mental health, engagement, health care, mental healthcare, mental well-being, mhealth, mobile health, mobile phone, randomized controlled trial, self-management, suicide prevention, telemedicine
The Purple Cluster	Covid-19 pandemic, emotion, empathy, resilience, schizophrenia, therapy, virtual agents

**Figure 8:**
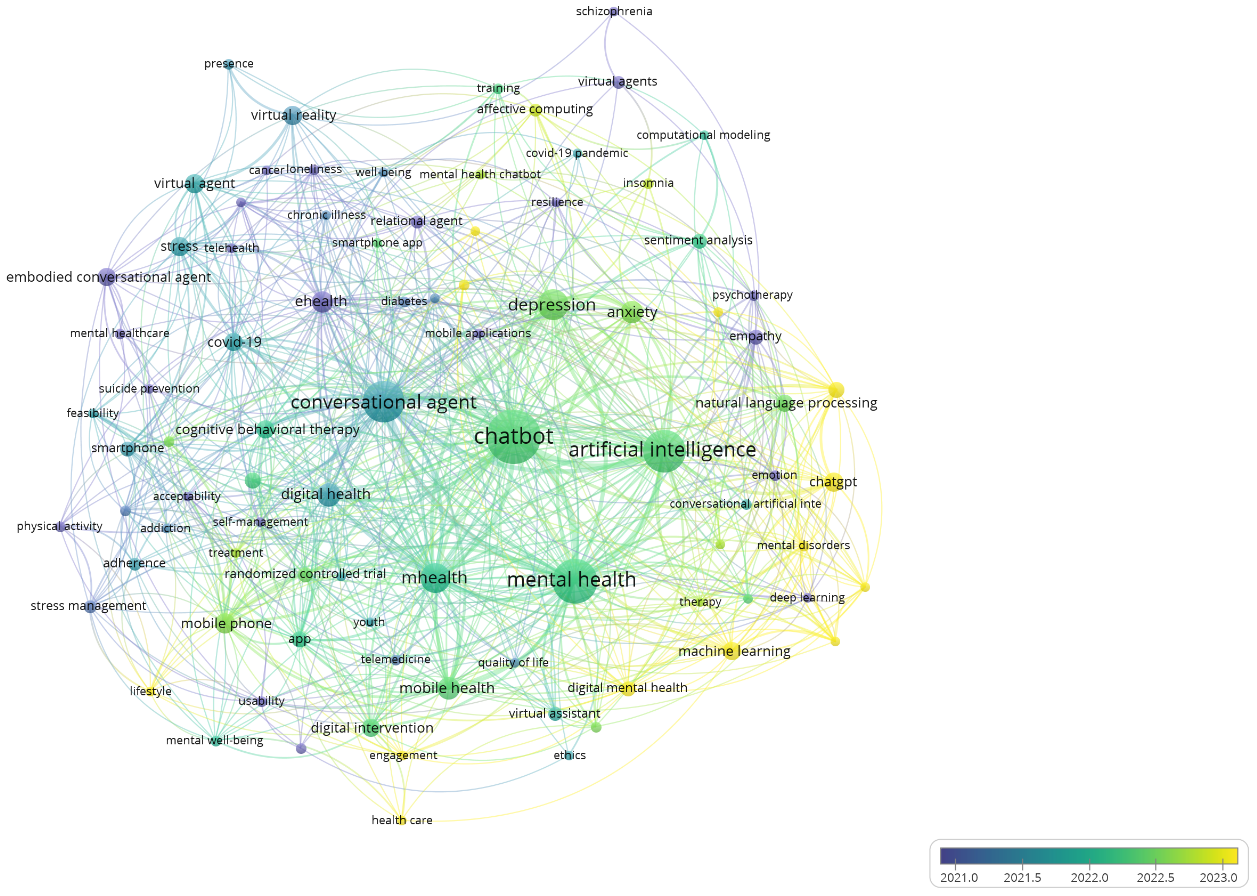
Time-based keyword co-occurrence network.

**Figure 9:**
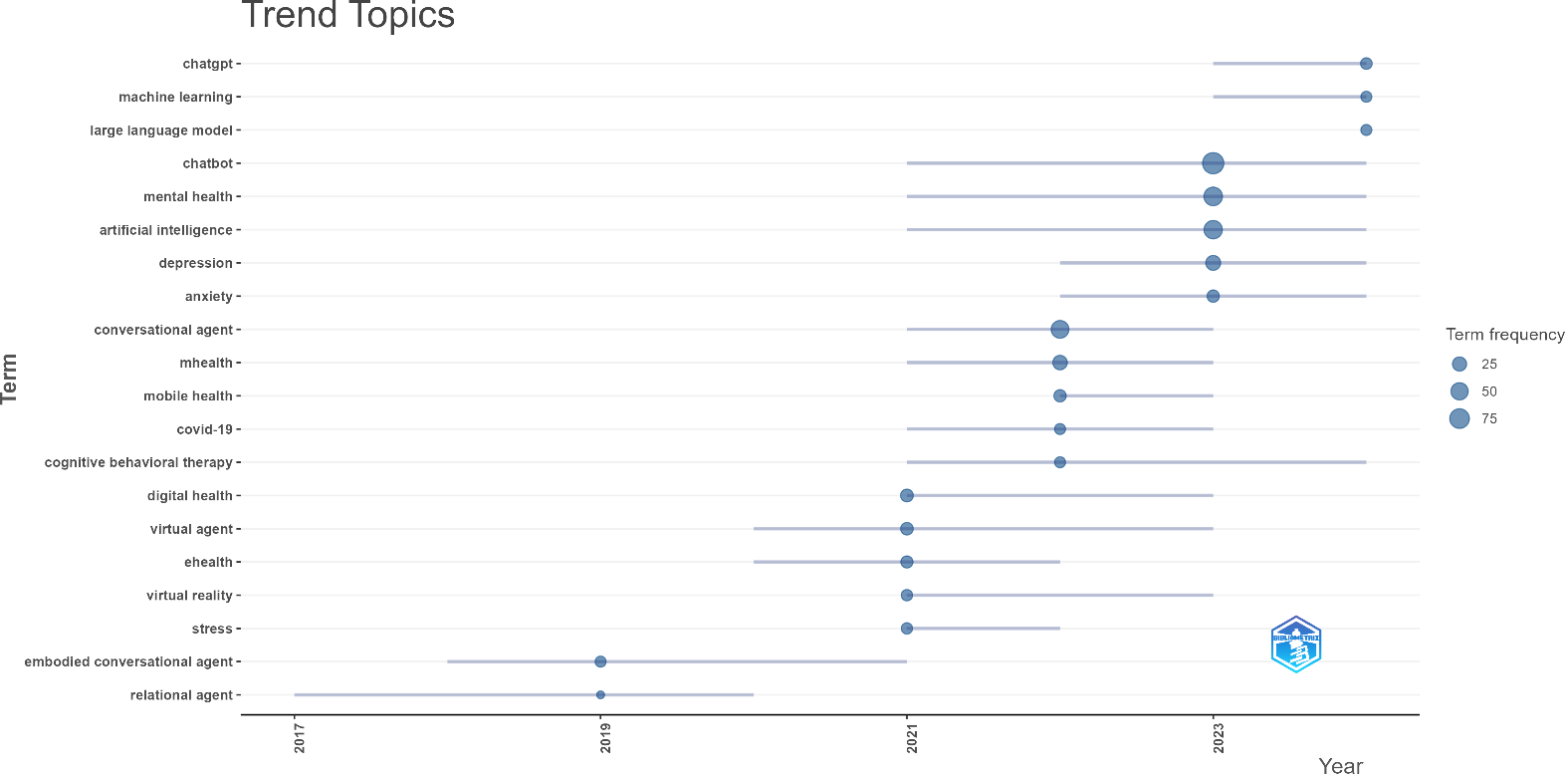
Top keywords utilized by the authors from 2015 to 2024.

In addition to analyzing the research topics, the temporal trends and hotspots of this research field could be analyzed by visualizing a time-based keyword co-occurrence network and exploring the keywords utilized by the authors during different periods. A total of 925 author’s keywords were identified across 261 articles and the results were depicted in **Figures 8** and **9**. An examination of the early research hotspots in this field reveals that the primary areas of focus were ‘virtual agents’ and ‘smart phone’ for addressing mental health concerns, including stress management, suicide prevention, and depression. Around 2021, the research hotspots shifted toward depression, anxiety, and cognitive behavioral therapy via digital mental health such as conversational agents, chatbot, mobile health, and artificial intelligence. By around 2023, keywords like ‘ChatGPT’, ‘machine learning’, and ‘large language models (LLM)’ represented recent research trends, suggesting that the latest technology in artificial intelligence has been introduced to the research or practical field of chatbots in mental health.

## 4 Discussion

### 4.1 Principal findings

This study conducted a bibliometric analysis on utilization of chatbots in mental health from 2015 to 2024. Over the last decade, research on utilization of chatbots in mental health has appeared to be increasing steadily. This field has undergone significant evolution and refinement, presently undergoing a period of accelerated expansion. Following 2019, notably during the pandemic’s peak in 2021, the landscape witnessed a notable increase in publications, accompanied by the emergence of novel and multifaceted subjects.

The United States have made significant and substantial contributions to the advancement and growth of publications in this research field. Additionally, England, Australia, China, France, Germany, Netherlands, Spain, Singapore, and Canada have demonstrated a high interest as well. National Center for Scientific Research in France is the most influential institution, and the most vigorous institutes are Imperial College London, University of Zurich, Nanyang Technological University, and National University of Singapore. The research on utilization of chatbots in mental health is predominantly favored by journals related to mental health or digital health, as indicated by the article counts and citation counts. JMIR Mental Health and Journal of Medical Internet Research are the most influential and productive publication sources in terms of average citations per article and articles counts. Collaboration among universities in the USA, United Kingdom, Switzerland, and Singapore demonstrated a high level. Global collaboration primarily focuses on the Americas, Europe, and Asia, with limited engagement from developing countries and even fewer contributions from underdeveloped regions. Consequently, it is essential to enhance collaboration in this field, incorporating both international and interdisciplinary partnerships to foster greater diversity of perspectives and expertise.

Through the cluster and evolutionary analyses conducted over time, key hotspots and development trends in this field were elucidated. Furthermore, the keyword co-occurrence network analysis revealed the prominent chatbot techniques characteristic of this multidisciplinary area, and highlighted a notable overlap between the identified clusters. Meanwhile, research on utilization of chatbots in mental health appeared to be dispersed within different cluster. A timeline of clusters revealed how this field evolved from earlier chatbots such as conversational agent and virtual agent to diverse and more advanced chatbots such as ChatGPT-based and AI-powered chatbots. Our keyword analysis revealed that the most prevalent mental health concerns associated with the widespread adoption of chatbots were anxiety, depression, suicidal ideation, and insomnia. As suggested by the keywords provided by the authors, “machine learning”, “deep learning”, and “natural language processing” have made a substantial impact on mental health. The current research landscape is characterized by a high frequency of keywords such as “ChatGPT”, “artificial intelligence”, and “large language models”, which reflect the latest advances and frontiers in the field. In essence, these findings highlight the need for continued investigation and exploration in this area, with the ultimate goal of advancing AI-based research in the development of mental health chatbots.

### 4.2 Future directions and challenge

Based on current research trends, technologies associated with artificial intelligence, including machine learning, natural language processing, and large language models, have become integral components of chatbot design. The integration of AI-powered mental health chatbots presents a promising avenue for addressing the growing demand for effective symptom management and augmenting therapeutic interventions. Despite the ongoing mental health challenges faced by society, AI chatbots have the potential to alleviate some of this burden, particularly in an era marked by a shortage of mental health professionals (35). However, it is essential to recognize that AI chatbots should be viewed as supplementary tools, rather than substitutes for human mental health professionals, whose expertise and empathy are indispensable in addressing complex mental health issues.

The focus of future research should be directed towards assessing the efficacy of AI-powered interventions, controlled trials, and methodologies for integrating AI chatbots into the clinical practice of mental health. AI-powered chatbots have demonstrated potential in providing accessible and convenient support, thereby addressing barriers to the help-seeking process for mental health issues. As AI technology continues to evolve and individuals become increasingly digitally connected, chatbots are likely to become more effective at delivering conversational and natural interactions, tailored to the unique needs and states of patients (18). Furthermore, the integration of AI chatbots into clinical practice may enable them to exhibit empathetic, curious, and understanding responses, facilitating collaborative interactions that can foster deeper patient motivation and adherence to treatment.

### 4.3 Limitations

One notable limitation of this study is that it was restricted to a single database for the identification of relevant studies, potentially leading to the exclusion of studies that may have been pertinent to the research question. Although WoSCC is a widely utilized academic database globally, it is essential to acknowledge that other databases specializing in medical research may offer more comprehensive and relevant information. To enhance the rigor and comprehensiveness of future studies, it is recommended that researchers conduct searches in a range of commonly utilized databases, including Scopus and Google Scholar, to ensure a more thorough analysis of the literature.

Another limitation of this analysis is that it solely relies on journal articles, thereby excluding other pertinent publication types in the field, such as review articles, book chapters, and conference proceedings. This limitation may impact the comprehensiveness of the results, particularly in terms of summarization and generalization. Furthermore, the predominantly English-language corpus may introduce a bias in the analysis, as it overlooks papers published in other languages. In contrast to topic modeling techniques, keyword co-occurrence analysis does not provide information on the top documents or the number of associated documents within each cluster. Therefore, future research could undertake topic modelling study to obtain deeper and comprehensive knowledge in this field.

## 5 Conclusion

This comprehensive bibliometric analysis provides an in-depth examination of the most prominent countries, institutions, journals, collaboration patterns, and research topics related to the utilization of chatbots in mental health over the past decade. The findings of this study offer a nuanced understanding of the intersection of chatbots and mental health, shedding light on the scientific patterns that underpin this emerging field on a global scale. Our research reveals that chatbots have a significant impact on promoting mental health, with applications ranging from addressing various mental health concerns at different stages to enhancing accessibility of mental health services and support for individuals. By leveraging chatbots, mental health professionals can provide targeted assistance to users, improving treatment outcomes for conditions such as depression, stress, and anxiety disorders through therapy, training, and counseling. As a valuable resource for mental health experts without a technology background and individuals interested in the application of chatbots in mental health, this study provides a comprehensive framework for understanding the potential benefits and limitations of this technology. In conclusion, the findings of this study offer a rich source of insights that can inform digital mental health practice, providing a solid foundation for future research and in-depth exploration of this complex and rapidly evolving field.

## Data Availability

The original contributions presented in the study are included in the article/supplementary material. Further inquiries can be directed to the corresponding author.
